# Transglutaminase Crosslinking Enhances Functional and Structural Properties of Fish Gelatins

**DOI:** 10.3390/polym17212822

**Published:** 2025-10-23

**Authors:** Sefik Tekle

**Affiliations:** Department of Food Engineering, Faculty of Engineering and Architecture, Kirsehir Ahi Evran University, 40100 Kirsehir, Türkiye; sefiktekle@ahievran.edu.tr; Tel.: +90-530-3273643

**Keywords:** fish gelatins, transglutaminase modification, rheological properties, emulsion and foam functionality, structural and thermal characterization

## Abstract

Fish gelatins are increasingly recognized as sustainable biopolymers for food, packaging, and biomedical applications; however, their functional performance often requires improvement. In this study, the effects of transglutaminase (TG) modification on the physicochemical and structural properties of trout (T) and sea bass (SB) gelatins were systematically investigated. TG treatment enhanced gel strength in a species- and concentration-dependent manner, with trout increasing from 100 g (control) to 108 g at 0.04% TG and SB reaching a maximum of 163 g at 0.01% TG. Rheological measurements confirmed improved viscoelastic behavior, particularly in trout samples (G′ > G″). Emulsifying activity was optimized at 0.02–0.04% TG in trout, while excessive cross-linking reduced interfacial activity; nevertheless, emulsion stability was improved in both species. Foaming capacity and stability reached 195% and 148%, respectively, in trout, whereas higher TG concentrations led to reductions in SB foaming performance. Scanning electron microscopy revealed denser and more homogeneous networks in TG-modified gels, correlating with their enhanced rigidity. Transparency remained high, while zeta potential shifted toward more negative values, indicating improved colloidal stability. FTIR, UV–Vis, and DSC analyses confirmed conformational rearrangements and thermal stabilization after cross-linking. Minor decreases in oil-binding capacity and slight color changes were also observed. Overall, TG cross-linking significantly enhanced the functional and structural properties of fish gelatins in a source-dependent manner, supporting their potential as versatile and sustainable biopolymers for diverse industrial applications.

## 1. Introduction

Gelatin is a natural biopolymer derived from the partial hydrolysis of collagen, widely utilized in the food, biomedical, and pharmaceutical industries due to its functional properties such as film formation, gelation, and emulsification [1]. Conventionally produced from bovine and porcine sources, gelatin’s use has raised religious, cultural, and health-related concerns, thereby driving the search for alternative sources [2]. In this context, fish-derived gelatins have gained increasing attention owing to their sustainability and promising functional characteristics [3].

However, the limited content of proline and hydroxyproline in fish gelatins results in low gel strength, reduced thermal stability, and poor mechanical resistance, thereby restricting their potential applications [4,5]. To overcome these limitations, several modification strategies have been developed [6,7]. Among these, enzymatic modification has emerged as a particularly promising approach [8]. Enzymatic techniques offer significant advantages in protein modification, including the production of structurally stable products, higher selectivity and substrate specificity, milder reaction conditions, and the use of sustainable enzyme sources [9]. Among enzymatic approaches, covalent cross-linking mediated by transglutaminase (TG), an acyltransferase, has demonstrated remarkable safety and efficiency [10]. TG catalyzes the formation of ε-(γ-Glu)-Lys isopeptide bonds between the γ-carboxamide groups of glutamine and the ε-amino groups of lysine residues within gelatin chains, thereby promoting cross-linking and enhancing the mechanical and functional properties of the gel network [11]. In recent years, various gelatins have been developed through TG-mediated modification of different sources, including bighead carp scales [12], commercial fish-type B [13], porcine skin-type A [14]; bovine bone, porcine skin, and cold-water fish skin [9], tilapia skin-type A [15], and commercial fish gelatin derived from tilapia skin [16]. These studies consistently confirm that TG modification significantly improves the physicochemical, functional, viscoelastic, thermal, foaming, and emulsifying properties of gelatins.

Rainbow trout (*Oncorhynchus mykiss*) and European sea bass (*Dicentrarchus labrax*) represent the two most extensively farmed fish species in Türkiye. According to TUIK statistics, national production in 2023 reached 222,486 tons for trout and 160,802 tons for sea bass [17]. In our previous studies, gelatin was extracted from the skins of these species, and their physicochemical properties and structural characteristics were comprehensively evaluated [18,19]. However, to date, no reports have been found in the literature addressing the modification of gelatin derived from trout and sea bass skins. Therefore, in order to broaden the potential applications of gelatin derived from the skins of these two major aquaculture species, the present study comprehensively investigated the physicochemical and structural properties of TG-crosslinked trout and sea bass gelatins. The analyses included gel strength, emulsifying activity index (EAI), emulsion stability index (ESI), foaming capacity, and stability, oil-binding capacity, rheological behavior, zeta potential, UV–Vis absorption spectra, Fourier transform infrared (FTIR) spectroscopy, differential scanning calorimetry (DSC), microstructural characterization (SEM), transparency, and color parameters.

## 2. Materials and Methods

### 2.1. Materials

Transglutaminase (TG, 120 U/g, microbial source: *Streptomyces mobaraensis*, purity: ≥95%) was purchased from Benosen Gida San. Kimya Ltd. Sti (İstanbul, Türkiye), phosphate-buffered saline (PBS, 0.01 M, pH: 7.4), sodium dodecyl sulfate (SDS), and the other chemicals were provided by Sigma-Aldrich (St. Louis, MO, USA).

### 2.2. Productions of Fish Skin and TG-Modified Gelatins

#### 2.2.1. Production of Fish Skin Gelatin

Sea bass and trout skins were obtained from Kilic Deniz A.S. (Mugla, Türkiye) as by-products of the filleting process. The skins were delivered to the laboratory in ice-packed containers. Only skins free of scales and residual muscle tissue were used. These skins were thoroughly washed with cold tap water to remove any remaining impurities and subsequently drained. The cleaned skins were stored at −20 °C for less than two months until further processing. Prior to gelatin extraction, the frozen skins were thawed in a refrigerator at 4 °C and cut into approximately 2–3 cm^2^ pieces.

Gelatin extraction was performed following the method described by Boran and Regenstein [20], with minor modifications. Initially, the fish skins were treated with an alkaline solution of NaOH (0.55 N, 5:1 *v*/*w*, pH: 11.82) for 67.5 min, followed by an acid treatment with HCl (0.1 N, 5:1 *v*/*w*, pH: 3.01) for 45 min. After each treatment, the skins were thoroughly washed with distilled water (5:1 *v*/*w*). Gelatin extraction was then carried out in a water bath using pure water (4:1 *v*/*w*) at 50 °C for 3 h (Memmert WNB45, Schwabach, Germany). The resulting gelatin solutions were collected in glass containers and dried at 60 °C for approximately 72 h. The final gelatin sheets had a moisture content of 5.48% and were stored in polyethylene bags in a dry environment until further use.

#### 2.2.2. Preparations of TG-Modified Fish Gelatins

Gelatin solutions were prepared at a concentration of 6.67% (*w*/*v*) by dissolving trout (T) and sea bass (SB), gelatins in distilled water at 50 °C under continuous stirring (350 rpm) for 60 min. The pH of each solution was subsequently adjusted to 7.5 using 1 M NaOH. Transglutaminase (TG) was then incorporated at concentrations of 0.01%, 0.02%, 0.04%, 0.06%, and 0.08% (*w*/*v*), and the mixtures were vortexed for 1 min to ensure homogeneity. Following this, the solutions were stirred at 50 °C (350 rpm) for 1 h to allow enzymatic crosslinking, after which the enzyme was inactivated by incubating the mixtures in boiling water for 10 min. The resulting TG-modified gelatin solutions were subsequently freeze-dried under vacuum to obtain dry, sponge-like, crystalline gelatin sheets. Ten distinct batches (T-TG0.01%, T-TG0.02%, T-TG0.04%, T-TG0.06%, T-TG0.08%, SB-TG0.01%, SB-TG0.02%, SB-TG0.04%, SB-TG0.06%, SB-TG0.08%) were prepared for further characterization and analyses.

### 2.3. Functional Properties of Fish and TG-Modified Gelatins

#### 2.3.1. Gel Strength

Gelatin hydrogel solutions were prepared at a concentration of 6.67% (*w*/*v*) by dissolving the gelatin in a water bath at 60 °C for 30 min. The solutions were then poured into Bloom jars and allowed to mature at 4 °C for 12 h to ensure gel formation. Bloom strength was determined using a Texture Analyzer equipped with a 5 kg load cell and a 1.27 cm cylindrical probe. The maximum force recorded during penetration was reported as the Bloom value in grams, following the method described by Boran and Regenstein [20].

#### 2.3.2. Emulsion Activity and Stability

The emulsion activity index (EAI) and emulsion stability index (ESI) of lyophilized fish and TG-modified gelatins were determined following the method of Zamorano-Apodaca et al. [21] with minor modifications. Briefly, 9 mL of a 0.1% (*w*/*v*) gelatin solution was mixed with 3 mL of sunflower oil and homogenized using an Ultra-Turrax (Daihan HG-15D, Seoul, Republic of Korea) at 18,000× *g* for 1 min. Immediately after homogenization (0 min) and 10 min later, 50 µL samples were collected from the bottom of the emulsion and diluted to 5 mL using 0.1% (*w*/*v*) sodium dodecyl sulfate (SDS). The absorbance of the diluted emulsions was measured at 500 nm using a UV–Vis spectrophotometer (Thermo Scientific GENESYS 10S, Waltham, MA, USA). The absorbance values recorded at 0 min (A_0_) and 10 min (A_10_) were subsequently used to calculate the EAI and ESI according to standard equations:

EAI (m^2^/g) = (2 × 2.303 × A_0_)/(C × φ) (1)

A_0_ = Absorbance at 500 nm wavelength

C = Initial protein concentration (g/mL)

Φ = Oil volume in emulsification.

ESI (min) = (A_0_ × Δt)/ΔA (2)

A_10_ = Absorbance at the end of 10 min.

Δt = 10 min.

ΔA = A_0_ − A_10_.

#### 2.3.3. Foam Capacity and Stability

Foam capacity and stability of lyophilized fish, and TG-modified gelatins were evaluated with minor modifications to the method of Zamorano-Apodaca et al. [21]. Briefly, 10 mL of a 0.5% (*w*/*v*) gelatin solution was homogenized at 14,000× *g* for 30 s at room temperature using an Ultra-Turrax device (Daihan HG-15D, Seoul, Republic of Korea). The total foam volume was recorded immediately after homogenization (0 min) and again after 10 min. Foam capacity was defined as the relative increase in foam volume at 0 min, whereas foam stability was determined based on the foam volume retained at 10 min. Foam expansion was calculated using the following equation:

Foaming expansion (%) = [(A − B)/B] × 100.(3)

A: Volume at different times (mL);

B: Volume before homogenization (mL).

#### 2.3.4. Oil-Binding Capacity (OBC)

The oil-binding capacity of lyophilized fish and TG-modified gelatins was assessed with slight modifications to the method described by Karoud et al. [22]. Approximately 50 mg of each sample was accurately weighed into pre-tared centrifuge tubes. Subsequently, 2 mL of sunflower oil was added, and the mixtures were incubated at room temperature for 1 h. During incubation, the contents were vortexed for 5 s at 15-min intervals using a Velp ZX3 Vortex Mixer (Velp, Usmate Velate, Italy) to ensure uniform dispersion. After incubation, the samples were centrifuged at 10,000× *g* for 15 min using a benchtop centrifuge (Andreas Hettich GmbH & Co. KG, Tuttlingen, Germany). The supernatant oil layer was carefully removed, and OBC was calculated as the volume of oil retained per gram of protein. A blank control containing only sunflower oil under identical conditions was used for reference.

### 2.4. Physicochemical Properties of Fish and TG-Modified Gelatins

#### 2.4.1. Zeta Potential

A 10% (*w*/*v*) solution of fish gelatins, both unmodified and modified with transglutaminase (TG), was prepared using PBS (pH: 7.4). The solutions were allowed to dissolve completely by incubation at 45 °C for 30 min in a thermostatically controlled water bath (Memmert WNB45, Schwabach, Germany). Zeta potential analyses were performed using a Zetasizer Nano ZSP (Malvern Instruments, Malvern, UK). For each sample, the reported zeta potential value represents the average of three independent replicates, with each replicate consisting of no fewer than 15 individual measurements [18].

#### 2.4.2. Transparency and Color Characteristics

Hydrogel solutions were prepared at a concentration of 1 mg/mL and equilibrated at 25 °C. The transmittance at 600 nm was measured using a spectrophotometer, and the transparency values were calculated according to the equation described by Kim et al. [23]:

Transparency (%) = 10^-transmittance at 600nm^ × 100 (4)

The color of fish gelatins, both unmodified and modified with transglutaminase (TG), was evaluated using a Chromameter (CR-100, Konica Minolta, Tokyo, Japan). Lightness (L*), redness (a*), and yellowness (b*) were recorded, with L* values ranging from 0 (black) to 100 (white), a* indicating red (+) to green (−), and b* representing yellow (+) to blue (−). The overall color difference (∆E*) was calculated following the method described by Tekle et al. [24].

ΔE* = [(ΔL*)^2^ + (Δa*)^2^ + (Δb*)^2^]^1/2^ (5)

Here, ΔL*, Δa*, and Δb* are the differences in L*, a*, and b* between the gelatins and TG-modified gelatins. L*, a*, and b* values of each sample were measured three times.

### 2.5. Rheological Properties of Fish and TG-Modified Gelatin Gels

The rheological properties of fish gelatin gels, both unmodified and modified with transglutaminase (TG) at a concentration of 6.67%, were characterized using a stress- and strain-controlled rheometer equipped with a Peltier temperature system (Anton Paar MCR302, Graz, Austria). A PP25 plate geometry was employed for frequency sweep measurements, maintaining a 1 mm gap between the plates. Analyses were performed at 5 °C under a strain of 0.5%, across an angular frequency range of 0.1–10 rad/s [25].

### 2.6. Structural Properties of Fish and TG-Modified Gelatins

#### 2.6.1. Molecular Weight Distribution (SDS–PAGE Analysis)

SDS–PAGE analysis was performed according to the method described by Bozkurt et al. [26] with slight modifications. Separation gels (7.5% and 20%) and stacking gels (4%) were prepared. Freeze-dried hydrolysate samples were mixed (1:1, *v*/*v*) with loading buffer containing 0.5 M Tris-HCl (pH 6.8), glycerol, 10% SDS, 0.5% bromophenol blue, and β-mercaptoethanol. The mixtures were boiled for 5 min prior to electrophoresis. Aliquots of 20 μL were loaded into each well, and electrophoresis was conducted using a vertical electrophoresis unit (Mini-PROTEAN^®^ System, Bio-Rad, Hercules, CA, USA) under a constant current of 20 mA per gel. Protein bands were visualized by staining with a solution of Coomassie Brilliant Blue (25%), methanol (50%), and acetic acid (50%), followed by destaining in a solution containing methanol (50%) and acetic acid (10%). Gels were imaged using a gel documentation system (Gel Doc EZ, Bio-Rad, Marnes-la-Coquette, France).

#### 2.6.2. FTIR Spectroscopy Analysis

A 10% (*w*/*v*) solution of fish gelatins, both unmodified and transglutaminase (TG)-modified, was prepared using deionized water. To ensure complete homogenization, the solutions were incubated at 45 °C for 30 min in a thermostatically controlled water bath (Memmert WNB45, Schwabach, Germany). ATR-FTIR spectra were recorded using a Bruker Tensor 27 spectrometer (Ettlingen, Germany) with a resolution of 4 cm^−1^ and 16 scans per spectrum in the mid-infrared region ranging from 4000 to 600 cm^−1^. Prior to each measurement, the crystal surface was cleaned with distilled water followed by 100% ethanol. For each sample, three spectra were obtained under identical conditions and subsequently averaged. Deconvolution analysis of all spectra was carried out by evaluating variations within the overlapping amide I region (1600–1700 cm^−1^) using OriginPro 2024 software [24]. Each sample was analyzed in triplicate to ensure reproducibility.

#### 2.6.3. UV-VIS Absorption Spectra

The UV–VIS absorption spectra of fish gelatins, both unmodified and transglutaminase (TG)-modified, were recorded using a UV–VIS spectrophotometer (Thermo Scientific GENESYS 10S, USA). Samples were prepared at a concentration of 1 mg/mL in distilled water, with distilled water serving as the blank reference. Spectra were collected over a wavelength range of 190–400 nm, at a resolution of 0.5 nm [27].

#### 2.6.4. Differential Scanning Calorimetry (DSC) Analysis

The thermal properties of fish gelatins, both unmodified and transglutaminase (TG)-modified, were evaluated using a DSC instrument (TA Instruments Q100, New Castle, DE, USA). Approximately 15 mg of each hydrogel sample was placed in aluminum pans and hermetically sealed. Samples were heated from 30 to 180 °C at a rate of 10 °C/min under a nitrogen atmosphere. Thermograms were recorded and analyzed using the accompanying software provided with the DSC instrument.

#### 2.6.5. Scanning Electron Microscopy (SEM) Analysis

The microstructural features of unmodified and transglutaminase (TG)-modified fish gelatins were examined using a field-emission scanning electron microscope (FESEM, Thermo Scientific Apreo 2 S LoVac, Waltham, MA, USA). Prior to observation, samples were affixed to carbon adhesive stubs and sputter-coated with a thin gold alloy layer under an argon atmosphere (20 mA current, 10^−3^ mbar) to improve surface conductivity. Micrographs were recorded at a magnification of 1000×.

### 2.7. Statistical Analysis

All experiments were conducted in triplicate. Data were analyzed by one-way analysis of variance (ANOVA) using JMP Pro 18 software (SAS, Cary, NC, USA), and mean comparisons were performed with Tukey’s multiple range test. Differences were considered statistically significant at *p* < 0.05.

## 3. Results and Discussion

### 3.1. Functional Properties of Fish and TG-Modified Gelatins

#### 3.1.1. Gel Strength

**Figure 1:**
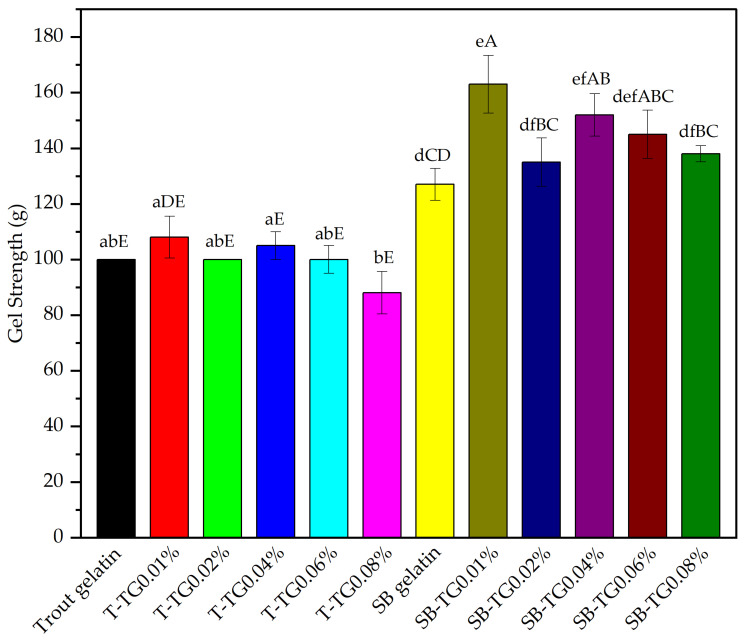
Gel strength of trout, sea bass and TG-modified gelatins. Error bars denote standard deviation (n = 3). Means with different lowercase letters indicate the statistical differences (*p* < 0.05) within the gelatin and TG-modified gelatins themselves, and different uppercase letters indicate statistical differences between all samples (*p* < 0.05). T: trout gelatin, SB: sea bass gelatin, TG: transglutaminase.

Gel strength is one of the most critical parameters for characterizing gelatin and assessing its suitability for various applications [28]. Analysis of the gel strength values obtained from trout (T) and sea bass (SB) gelatins treated with different concentrations of TG revealed pronounced structural differences between them (Figure 1). For trout gelatin, the gel strength of the control sample, measured at 100 g, and upon TG addition, only minor fluctuations within the range of 88–108 g were observed. This indicates that the strengthening effect of TG on the gel structure of trout gelatin is relatively limited, suggesting a lower susceptibility of proteins in this species to enzymatic cross-linking. In contrast, the gel strength of sea bass gelatin, which was 127 g in the control sample, increased to 163 g with the addition of 0.01% TG, representing the highest value observed in this study. As TG concentration increased, gel strength values were recorded as 135 g, 152 g, 145 g, and 138 g, indicating that the maximum effect was achieved at lower concentrations, whereas higher enzyme levels led to a plateau or slight reduction in gel strength due to enzyme saturation [7]. This finding demonstrates that TG induces cross-linking within the hydrogel network through non-disulfide covalent bonds, thereby enhancing the formation of TG-catalyzed covalent linkages [29]. Overall, the results indicate that sea bass gelatin is considerably more responsive to TG-mediated structural modification than trout gelatin, leading to the formation of stronger gel networks. The results indicate that the presence of higher molecular weight fractions or more favorable substrate sequences in sea bass gelatin may have contributed to the improved catalytic activity of TG. At a concentration of 0.01% TG, both gelatin samples exhibited their maximum gel strengths (108 g and 163 g), suggesting a noteworthy potential for application in functional food formulations. Similar improvements in gel strength following TG treatment have been reported for tilapia and New Zealand hoki gelatins [29,30]. Collectively, these findings demonstrate that the effect of TG on fish gelatin is highly dependent on both the gelatin source and the enzyme concentration applied.

#### 3.1.2. Emulsifying Activity and Stability

**Figure 2:**
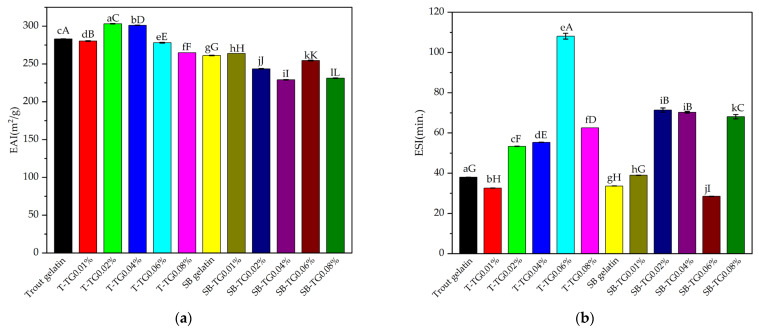
EAI (**a**) and ESI (**b**) of trout, sea bass and TG-modified gelatins. Error bars denote standard deviation (n = 3). Means with different lowercase letters indicate the statistical differences (*p* < 0.05) within the gelatin and TG-modified gelatins themselves, and different uppercase letters indicate statistical differences between all samples (*p* < 0.05). T: trout gelatin; SB: sea bass gelatin; TG: transglutaminase.

EAI is an important functional indicator of the emulsifying capacity of proteins [1]. As shown in Figure 2a, trout gelatin exhibited the highest EAI (283.05 m^2^/g), confirming its strong surface activity. TG treatment further increased the EAI at low and moderate concentrations (T-TG0.02%: 303.20 m^2^/g; T-TG0.04%: 301.17 m^2^/g), whereas a significant reduction was observed at higher concentration (T-TG0.08%: 264.94 m^2^/g), likely due to excessive cross-linking that limited surface-active sites (*p* < 0.05). In contrast, sea bass gelatin demonstrated a lower EAI in the control group (261.25 m^2^/g), with TG treatment providing only marginal improvements, followed by a pronounced decrease in EAI at elevated concentrations. Notably, the EAI values obtained in this study were considerably higher than those previously reported for bovine bone, porcine skin, and cold-water fish skin gelatins treated with TG [9]. These results indicate that TG enhances the emulsifying properties of fish gelatin at low levels by modulating protein conformation, but excessive cross-linking at higher levels compromises the accessibility of functional groups required for interfacial stabilization.

ESI is a quantitative indicator of the resistance of an emulsion to phase separation over time. Beyond emulsification capacity, the analysis also provides insights into the physical stability of the emulsion system, which can be directly influenced by protein structural stability, the degree of cross-linking, and viscoelastic properties [31]. The ESI values of trout and sea bass and their TG-modified gelatins are presented in Figure 2b. In the control group, trout gelatin exhibited a moderate ESI (38.00 min). Following TG treatment, emulsion stability significantly improved, with the highest value observed for T-TG0.06% (108.11 min). Other TG-treated groups (T-TG0.02%: 53.37 min; T-TG0.04%: 55.35 min; T-TG0.08%: 62.52 min) also showed significant increases compared to the control. In contrast, insufficient cross-linking at low enzyme concentration (T-TG0.01%) resulted in a reduced ESI (32.61 min). Sea bass gelatin displayed lower stability in the control group (33.63 min) compared to other treatment groups. However, TG treatment generally enhanced stability, particularly in SB-TG0.02% (71.36 min), SB-TG0.04% (70.25 min), and SB-TG0.08% (68.10 min). Conversely, SB-TG0.06% exhibited a decline in stability (28.51 min), likely due to excessive cross-linking. Overall, appropriate TG concentrations improved emulsion stability in both gelatin types. Comparable trends have also been reported for bovine bone, porcine skin, and cold-water fish skin gelatins treated with TG [9]. These findings further demonstrate that EAI and ESI are not fully correlated, as EAI reflects the initial droplet formation capacity, whereas ESI represents the long-term structural integrity of the emulsion (Figure 2a,b).

#### 3.1.3. Foam Capacity and Stability

**Figure 3:**
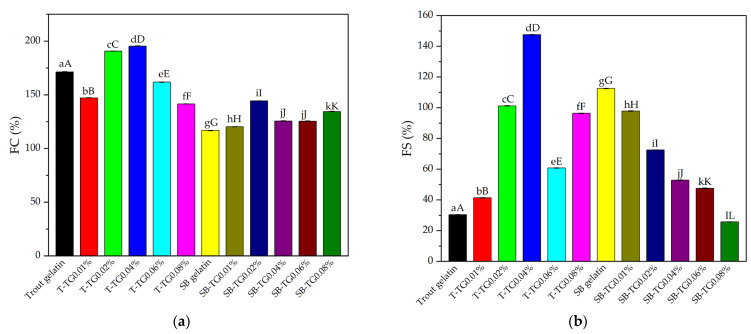
Foam capacity (**a**) and stability (**b**) of trout, sea bass and TG-modified gelatins. Error bars denote standard deviation (n = 3). Means with different lowercase letters indicate the statistical differences (*p* < 0.05) within the gelatin and TG-modified gelatins themselves, and different uppercase letters indicate statistical differences between all samples (*p* < 0.05). T: trout gelatin, SB: sea bass gelatin, TG: transglutaminase.

Foaming capacity (FC) is an important functional property that reflects the surface activity of proteins and their ability to stabilize the air phase, typically achieved by incorporating air into a protein solution through whipping, mixing, shaking, or sparging [9]. The ability of gelatin molecules to spread at the interface, undergo partial denaturation, and form films at the air–water boundary plays a decisive role in their foaming capacity [27]. TG-induced cross-linking can alter these interfacial properties. The FC values of trout and sea bass and their TG-modified gelatins are presented in Figure 3a. In the control group, trout gelatin exhibited an FC of 171.41%. Low TG concentrations (0.01–0.04%) enhanced this property, with the maximum value (195.35%) recorded at 0.04%. However, higher TG concentrations (0.06–0.08%) reduced FC, suggesting that excessive cross-linking restricted the molecular flexibility required for interfacial spreading. Sea bass gelatin displayed a lower initial FC (116.81%), which increased at low TG levels (up to 144.34%), while higher enzyme concentrations resulted in only marginal changes. Overall, TG treatment improved the foaming capacity of both gelatin types at low to moderate levels, whereas high concentrations exerted a negative effect (*p* < 0.05). These results are consistent with previous findings for bovine bone, porcine skin, and cold-water fish skin gelatins treated with TG [9], confirming that the effect of transglutaminase on protein functional properties is strongly dependent on both enzyme concentration and protein source.

Foam stability (FS) is one of the critical parameters determining the functional performance of protein-based hydrogel systems. The FS values of trout and sea bass and their TG-modified gelatins are shown in Figure 3b. In trout gelatin, the control group exhibited low stability (30.45%), which markedly increased to 147.50% with 0.04% TG treatment. This suggests that optimal cross-linking enhanced the ability of proteins to form stable interfacial films. Although higher TG concentrations (0.06–0.08%) resulted in a decline, FS remained above the control level. In contrast, sea bass gelatin displayed a relatively high initial FS (112.55%), but this value gradually decreased with TG addition, reaching 25.73% at 0.08% TG. Overall, the FS values observed in this study were generally higher than those reported for bovine bone, porcine skin, and cold-water fish skin gelatins subjected to TG treatment [9]. These findings demonstrate that the influence of TG on foam stability is strongly dependent on the gelatin source, and highlight the importance of identifying an optimal enzyme concentration to enhance the functional performance of gelatin-based systems.

#### 3.1.4. Oil-Binding Capacity (OBC)

Oil-binding capacity (OBC) is a key functional property of proteins, reflecting their ability to absorb oil and retain it within the protein matrix against gravitational forces [31]. In this study, the OBC of trout (T) and sea bass (SB) gelatins, as well as their TG-modified counterparts, varied depending on both the protein source and the enzyme concentration (Table 1). The highest OBC value was recorded in the trout control group (16.06 ± 0.25%). This value significantly decreased with TG treatment, reaching as low as 7.62% in the T-TG0.04% group (*p* < 0.05). Sea bass gelatin exhibited a lower initial OBC (8.97 ± 0.32%), and a similar decreasing trend was observed after TG modification, with the lowest value measured in SB-TG0.02% (6.80 ± 0.89%). Overall, TG application limited the OBC of both gelatin types, which may be attributed to the reduced availability of free hydrophobic regions caused by cross-linking [28]. These findings suggest that TG-modified gelatins may offer potential advantages in applications where a lower oil-binding capacity is desired.

### 3.2. Physicochemical Properties of Fish and TG-Modified Gelatins

#### 3.2.1. Zeta Potential

**Figure 4:**
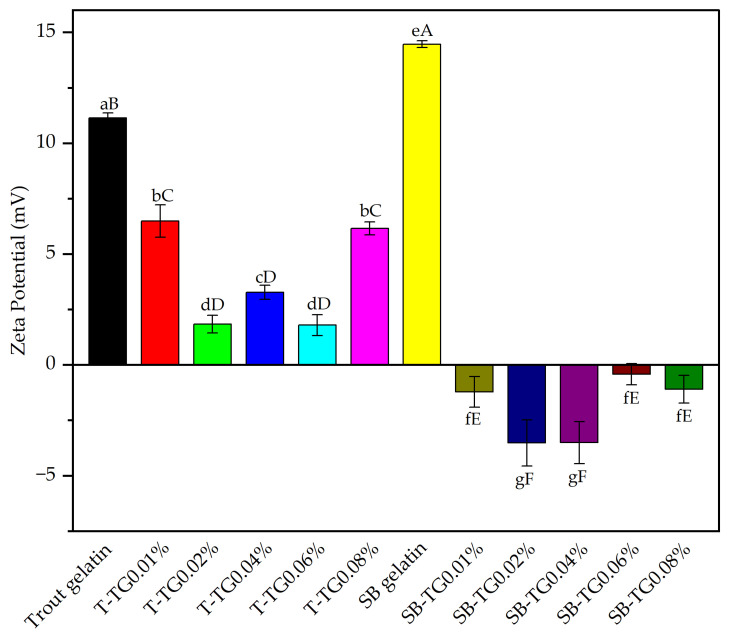
Zeta potential values of trout, sea bass and TG-modified gelatins. Error bars denote standard deviation (n = 3). Means with different lowercase letters indicate the statistical differences (*p* < 0.05) within the gelatins and TG-modified gelatins themselves, and different uppercase letters indicate statistical differences between all samples (*p* < 0.05). T: trout gelatin; SB: sea bass gelatin; TG: transglutaminase.

Zeta potential is a critical parameter that characterizes the surface charge of particles in a colloidal system and reflects the electrostatic stability of the dispersion. Generally, higher absolute values of zeta potential indicate greater system stability, as repulsive forces between particles help prevent aggregation [13]. The polarity of zeta potential, whether positive or negative, can be influenced by factors such as pH, protein structure, and chemical or enzymatic modifications. As shown in Figure 4, the differences in zeta potential between the control FG and TG-modified gelatin samples were statistically significant (*p* < 0.05). TG modification resulted in slightly positive values in trout gelatin, whereas in sea bass gelatin, the potential shifted from stable positive values to weakly negative ones, suggesting that TG predominantly interacted with positively charged surface groups in the latter [13]. Trout gelatin initially exhibited a positive charge (11.13 mV), indicating high colloidal stability. Upon TG treatment, a general reduction in zeta potential was observed, with notable decreases at 0.02% and 0.06% TG (1.40 and 1.79 mV, respectively). This decline indicates that the surface charges approached neutrality, leading to weakened electrostatic stabilization. In contrast, sea bass gelatin had a higher initial zeta potential (14.47 mV), but TG modification shifted the values into the negative range, particularly at 0.02–0.04% TG where the potential decreased to approximately −3.5 mV. This transition suggests that ionic groups on the protein surface were involved in cross-linking reactions, resulting in a redistribution of charge and a shift toward the isoelectric point. Overall, TG treatment reduced the zeta potential of both gelatin types, thereby lowering electrostatic repulsion between molecules. Comparable results have been observed in fish scale gelatin–high methoxyl citrus pectin gels following TG treatment [32]. Although this reduction could increase aggregation tendencies, the formation of covalent cross-links simultaneously contributed to reinforcing the gel network structure.

#### 3.2.2. Transparency and Color Characteristics

**Figure 5:**
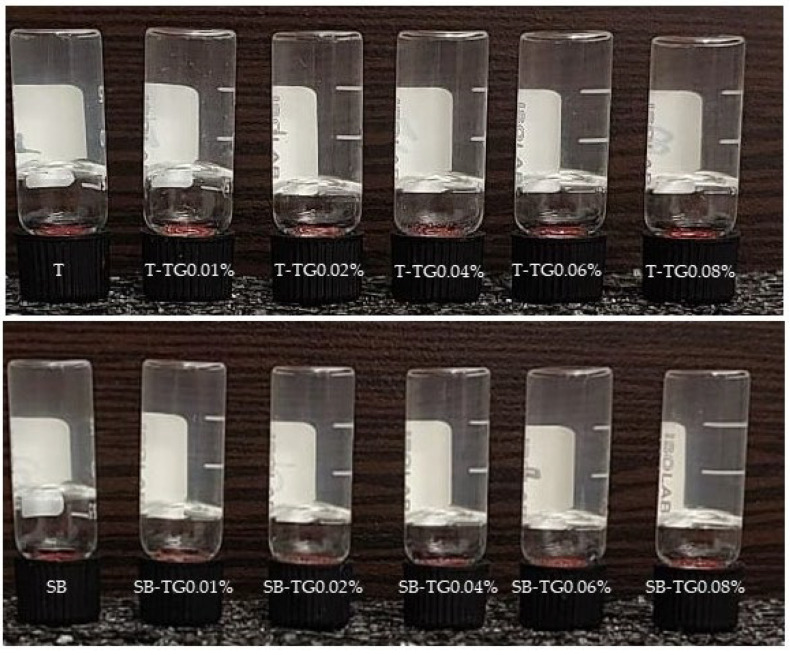
Trout, sea bass and TG-modified gelatin gels. T: trout gelatin; SB: sea bass gelatin; TG: transglutaminase.

The functional performance of fish gelatins is strongly influenced by both structural and sensory properties, including optical clarity and color, which are critical for food applications. In this study, trout and sea bass gelatins: unmodified and TG-modified, exhibited distinct transparency and color profiles (Table 1 and Table 2, Figure 5). Trout gelatin demonstrated high baseline transparency (99.61%), which increased to 100% following TG incorporation, representing a statistically significant improvement (*p* < 0.05). However, at 0.06% TG, transparency slightly decreased to 99.31%, likely due to excessive crosslinking and light scattering. Sea bass gelatin displayed a comparable control transparency (99.54%); with a minor increase at 0.01% TG (99.77%), but beyond 0.02% TG, transparency declined to 99.31% and remained stable, suggesting lower tolerance to TG-induced modifications. The reduced transparency in SB gelatin may be due to the TG modification process promoting the aggregation of proteins [9]. Similar results were obtained from TG-crosslinked fish gelatins by Lin et al. [33].

Regarding color, both gelatin types exhibited high L* values, indicating light inherent colors, with trout at 100.58 and sea bass at 100.19. TG incorporation caused a slight decrease in L* of trout gelatin, statistically significant at 0.06–0.08% TG (*p* < 0.05). Sea bass gelatin showed reductions at 0.02–0.04% TG, likely due to increased structural density. The a* values were negative in both types, reflecting greenish tones; TG shifted trout gelatin toward more neutral hues, whereas changes in sea bass gelatin were minimal. For b*, trout gelatin exhibited fluctuating increases, while sea bass gelatin showed a general upward trend, indicative of enhanced yellowness. ΔE values remained low in trout gelatin (1.37–2.30) but were more pronounced in sea bass gelatin at intermediate TG levels (2.69–2.74), decreasing at higher concentrations. Collectively, these findings indicate that TG modification influences both transparency and color in a dose-dependent manner, with trout gelatin maintaining high optical clarity over a wider concentration range, while sea bass gelatin reaches saturation earlier and exhibits greater yellowing.

### 3.3. Rheological Properties of Fish and TG-Modified Gelatin Gels

**Figure 6:**
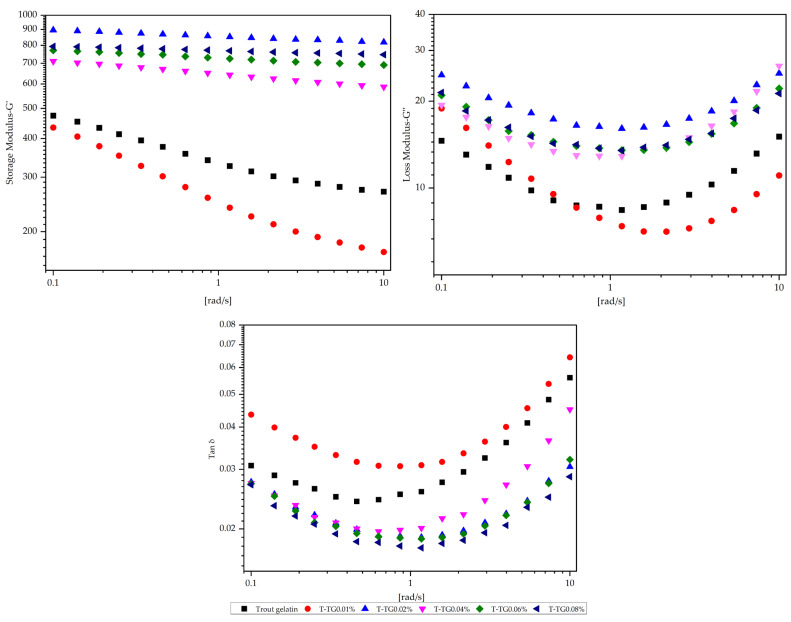
Rheological properties of trout and TG-modified gelatin gels. T: trout gelatin; TG: transglutaminase.

**Figure 7:**
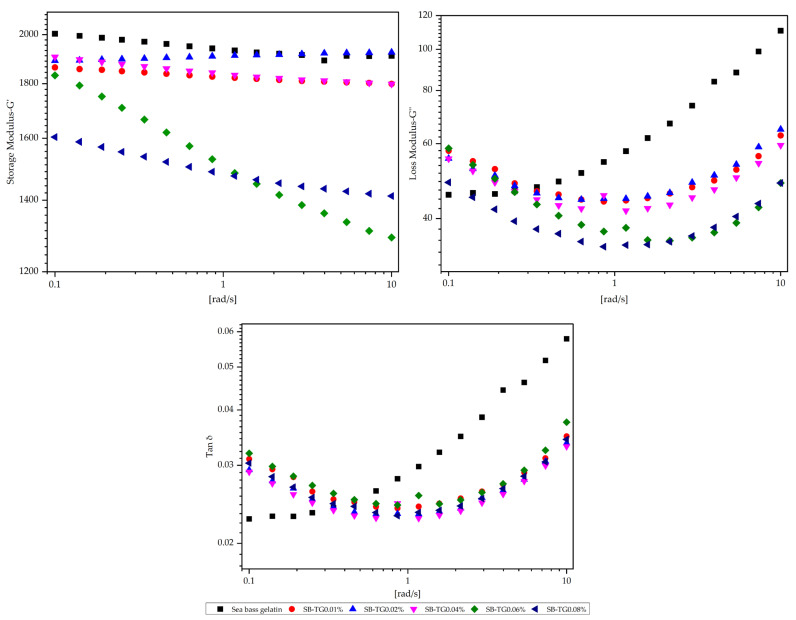
Rheological properties of sea bass and TG-modified gelatin gels. SB: sea bass gelatin; TG: transglutaminase.

Rheological analyses were conducted to evaluate the viscoelastic properties and gel network stability of fish gelatin gels, revealing pronounced structural differences induced by TG modification in both trout and sea bass gelatins (Figure 6 and Figure 7). Trout gelatin at low TG concentrations (T-TG0.01%) exhibited weak elastic networks, as indicated by the low storage modulus (G′) values, whereas the T-TG0.02% sample showed a maximum G′, reflecting enhanced crosslinking and improved gel network formation [34]. At higher TG levels (0.06–0.08%), further increases in G′ supported the development of stronger gel structures. In contrast, sea bass gelatin naturally formed a robust gel matrix, with high G′ in the control sample. Low TG concentrations (0.01%) reduced elastic properties, while intermediate concentrations (0.02–0.04%) resulted in the most balanced viscoelastic behavior. At higher concentrations (0.06–0.08%), excessive crosslinking led to decreased G′ and structural heterogeneity. For both gelatin types, G′ consistently exceeded the loss modulus (G″), confirming gelation and aligning with literature reports [13,29]. The positive correlation between G′ and the content of triple-helix-like structures in gelatin gels further suggests that reduced G′ at high TG levels reflects that the formation of fewer triple-helix-like structures occurs due to ε-(γ-Glu)-Lys isopeptide bond formation during gelation [8]. Although G′ is typically regarded as a rheological indicator of gel strength, the present results revealed no direct correlation between G′ and gel strength [33,35]. This discrepancy may arise from differences in measurement principles—dynamic oscillatory testing reflecting the viscoelastic network response under small deformations, while gel strength represents the macroscopic fracture behavior of the gel matrix. Overall, TG enhanced gel stiffness and network stability at optimal concentrations, whereas excessive TG weakened elastic properties, consistent with previous findings linking higher G′ to increased triple-helix content [29].

**Figure 8:**
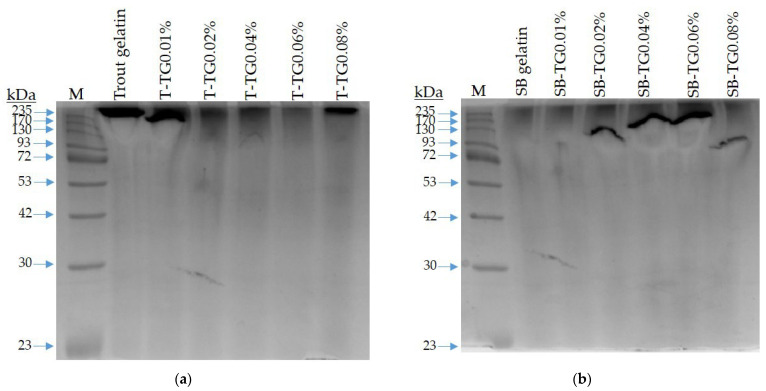
The SDS-PAGE patterns of trout (**a**), sea bass (**b**) and TG-modified gelatins. T: trout gelatin; SB: sea bass gelatin; TG: transglutaminase; M: marker.

**Figure 9:**
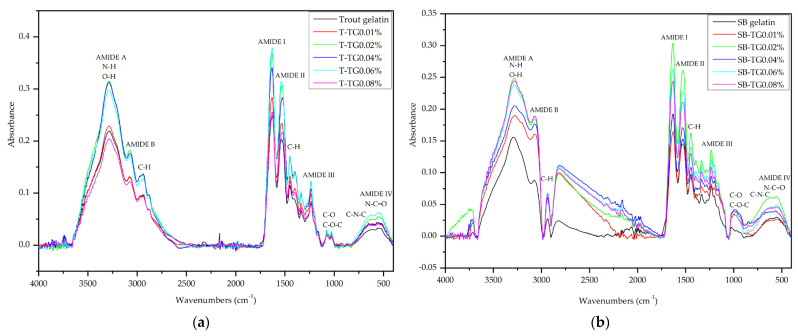
FTIR of trout (**a**), sea bass (**b**) and TG-modified gelatins. T: trout gelatin; SB: sea bass gelatin; TG: transglutaminase.

Tan δ (G″/G′) values obtained for trout and sea bass gelatin gels treated with varying TG concentrations provided additional insights into their viscoelastic behavior (Figure 8 and Figure 9). Values below 0.1 indicated strong, elastic-dominant networks [12]. TG treatment generally strengthened the elastic properties of both gelatin types; however, the effect was limited in trout gelatin, where low TG concentrations produced weaker elasticity, and high concentrations slightly reduced tan δ, although they remain higher than sea bass values. Sea bass gelatin exhibited low and stable tan δ values at TG concentrations of 0.04% and above, reflecting a dense, stable, and highly elastic network. These interspecies differences are likely attributable to variations in molecular characteristics and substrate sensitivity to TG. Consequently, sea bass gelatin appears more responsive to TG modification and demonstrates superior rheological performance for functional food applications. Comparable findings have been reported for bighead carp scale gelatin modified with TG and pectin [12].

### 3.4. Structural Properties of Fish and TG-Modified Gelatins

#### 3.4.1. Molecular Weight Distribution

The SDS-PAGE patterns (Figure 8a,b) of trout (T) and sea bass (SB) gelatins exhibited characteristic α- and β-chain distributions derived from the partial hydrolysis of collagen, where the α-chains (α_1_, α_2_) migrated within the 80–125 kDa range, and the β-chains were observed between approximately 160 and 250 kDa [36]. SDS-PAGE results indicated that the prominent band observed around 235 kDa in the trout gelatin sample likely corresponded to β-chains, representing partially cross-linked or aggregated gelatin subunits. Upon TG treatment, the intensity of this band decreased, accompanied by the appearance of faint, higher-molecular-weight regions. This suggests that TG catalyzed further cross-linking among β-chains, leading to the formation of larger, possibly insoluble macromolecular assemblies [30]. In contrast, sea bass gelatin initially exhibited distinct α- and β-chain bands with lower high-molecular-weight aggregation. Following TG modification, these bands gradually shifted toward higher molecular weights, indicating progressive formation of ε-(γ-Glu)-Lys linkages between peptide chains. The effect observed in TG-modified SB gelatin was consistent with the results obtained for the three types of TG-modified gelatins [9].

#### 3.4.2. FTIR Analysis

The effects of TG modification on the functional groups and secondary structures of fish gelatins were evaluated using FTIR spectroscopy (Figure 9a,b). Although minor spectral differences were observed among the gelatins, all samples exhibited prominent peaks in the amide regions. The Amide A band (~3280–3300 cm^−1^) appeared at 3284 cm^−1^ for trout gelatin and 3291 cm^−1^ for sea bass gelatin, with the latter indicating relatively weaker hydrogen bonding [37]. TG treatment induced slight shifts in both gelatin types, suggesting modest structural rearrangements. Limited changes were also observed in the Amide I (~1631–1633 cm^−1^) and Amide II (~1527–1536 cm^−1^) bands, indicating that the secondary structures were largely preserved, although structural reorganization was more pronounced in trout gelatin [18]. The Amide B and III bands remained largely unaffected, demonstrating that TG had minimal influence in these regions. Overall, the FTIR results indicate that TG induces subtle structural modifications in gelatin molecules while maintaining the integrity of the primary protein architecture [9].

Deconvolution of the amide I band revealed that TG treatment induced concentration-dependent alterations in the secondary structure of trout (T) and sea bass (SB) gelatins (Table 3). For T gelatin, the β-sheet fraction (40.69%) progressively declined to 37.23% at 0.02% TG, accompanied by an increase in random coil (15.98%) and α-helix (12.00%) contents, suggesting partial chain unfolding and enhanced molecular flexibility. At higher TG levels (0.08%), the β-sheet proportion further decreased to 29.46%, while β-turns rose markedly to 46.47%, indicative of excessive cross-linking and conformational relaxation [7]. In contrast, SB gelatin exhibited a more ordered native structure, characterized by higher initial β-sheet (53.38%) and α-helix (16.17%) contents. TG incorporation reduced β-sheet abundance (to ~40–46%) and increased β-turns (up to 38.18%), implying partial disruption of intermolecular hydrogen bonding and rearrangement of peptide chains. These structural transitions suggest that moderate TG concentrations promote ordered interchain association via ε-(γ-glutamyl)–lysine cross-links, whereas higher enzyme levels induce steric congestion and network loosening [9]. Collectively, the results indicate that TG-mediated conformational modification is both source- and concentration-dependent, with SB gelatin demonstrating higher structural integrity, while T gelatin exhibited greater conformational adaptability under enzymatic cross-linking [38].

#### 3.4.3. UV-VIS Absorption Spectra

**Figure 10:**
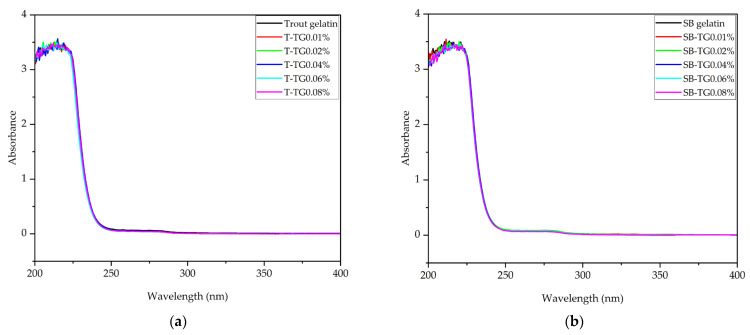
UV-Vis absorption spectrum of trout (**a**), sea bass (**b**) and TG-modified gelatins. T: trout gelatin; SB: sea bass gelatin; TG: transglutaminase.

UV–Vis spectroscopy was employed to assess the structural characteristics of trout and sea bass gelatins, both unmodified and TG-modified. In all samples, maximum absorbance was observed within the 200–230 nm range (Figure 10a,b), corresponding to n → π* transitions of peptide bonds in the protein backbone [27]. For trout gelatin, the highest absorbance was recorded at 215 nm (T-TG0.04%, 3.56), while the lowest absorbance occurred between 214.5 and 212 nm (T-TG0.06–0.08%, 3.48). Sea bass gelatin showed a maximum absorption at 211.5 nm (SB-TG0.01%, 3.54) and a minimum absorption between 220.5 and 211 nm (SB-TG0.06–0.08%, 3.45). The slight redshift in absorbance for trout samples suggests enhanced polypeptide interactions induced by TG, whereas sea bass samples displayed a wavelength shift accompanied by a modest decrease in absorbance. The rapid decline in absorbance between 230 and 260 nm indicates a low content of aromatic groups; and negligible absorbance above 270 nm reflects the limited presence of aromatic amino acids such as phenylalanine, tyrosine, and tryptophan [37]. Overall, TG modification induced only minor structural adjustments in both gelatin types, while the fundamental characteristics of peptide bonds remained largely preserved.

#### 3.4.4. Differential Scanning Calorimetry (DSC) Analysis

**Figure 11:**
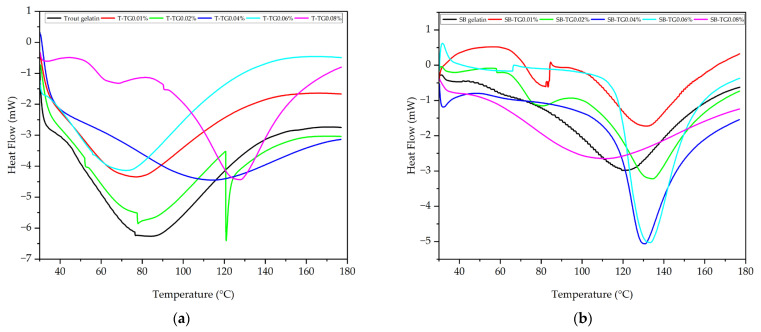
DSC properties of trout (**a**), sea bass (**b**) and TG-modified gelatins T: trout gelatin; SB: sea bass gelatin; TG: transglutaminase.

Differential scanning calorimetry (DSC) was employed to evaluate the effect of TG modification on the thermal stability of trout and sea bass gelatins (Figure 11a,b, Table 1). The denaturation temperature (Td) of gelatin typically corresponds to the helix-to-coil transition, representing the disruption of hydrogen bonds within triple-helix structures and the formation of random coils, thereby reflecting the stability of triple-helix-like domains in the gelatin matrix [8]. Trout gelatin initially exhibited a low Td (84.20 °C), which increased markedly upon TG treatment, particularly at 0.02% (120.95 °C) and 0.08% (127.70 °C), indicating the formation of a more stable triple-helix-like structure. However, at 0.06% TG, Td decreased to 72.24 °C, suggesting that excessive crosslinking induced structural irregularities. Sea bass gelatin displayed a higher baseline Td (120.89 °C), which further increased to 134.45 °C at TG concentrations of 0.02–0.06%, whereas at 0.08% TG, thermal stability decreased to 111.51 °C. These results demonstrate that TG enhances thermal resistance in both gelatin types, but exceeding the optimal concentration disrupts structural homogeneity and limits stability. Similar trends have been reported for TG-modified porcine Type A and bovine Type B gelatins [11]. Furthermore, decreases in G′ values have been associated with reductions in Td, highlighting a link between gel network integrity and thermal behavior [8]. Overall, TG treatment strengthens covalent crosslinking between protein chains, thereby enhancing the thermal stability of both trout and sea bass gelatins [27].

#### 3.4.5. Scanning Electron Microscopy (SEM) Analysis

**Figure 12:**
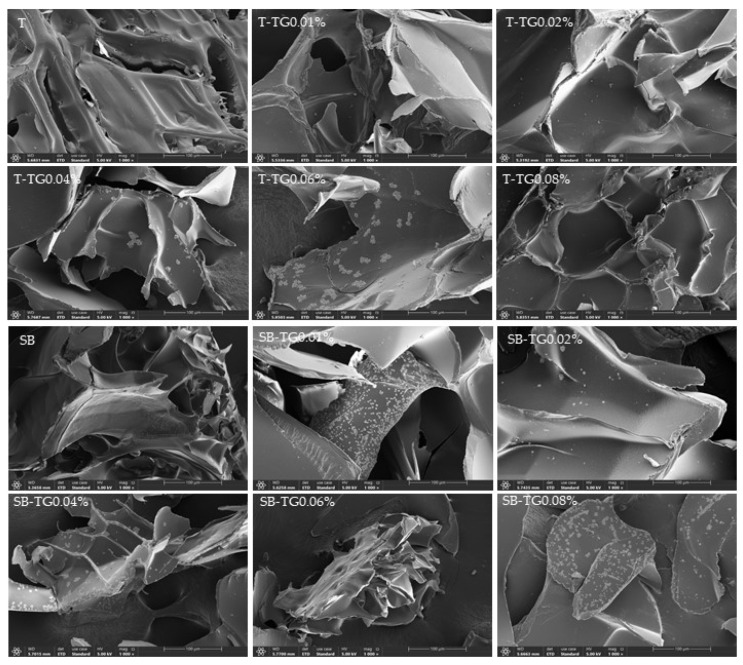
Microstructure properties of trout, sea bass and TG-modified gelatins (Magnification: 1000×). T: trout gelatin; SB: sea bass gelatin; TG: transglutaminase.

Scanning electron microscopy (SEM) was employed to investigate the microstructural features of trout and sea bass gelatins treated with varying TG concentrations. Compared to control samples, TG-modified gelatins exhibited denser and more homogeneous crosslinking, confirming the reinforcement of the gelatin network through TG-catalyzed covalent bonds (Figure 12). In trout gelatin, the control sample displayed a fibrous and relatively ordered structure, whereas transglutaminase (TG) incorporation increased surface smoothness and promoted the formation of a more compact network. Particularly at 0.06% and 0.08% TG, the gelatin network exhibited a denser, homogeneous, and porous morphology, indicating effective crosslinking. In contrast, sea bass gelatin control samples showed a looser, irregular, and fragmented network. TG treatment induced granular formations and irregular densities on the surface, and at higher TG concentrations (0.06–0.08%), a compact but heterogeneous structure was observed. Both control groups displayed fragmented networks with thin walls, likely due to insufficient hydrogen content required for crosslinking. This was a consequence of low proline and hydroxyproline concentrations, resulting in weaker gel networks [39]. Furthermore, SEM observations suggest that the increased microstructural density induced by TG correlates with enhanced gel strength, whereas excessive TG concentrations lead to irregular structures that weaken this correlation [28]. Overall, trout gelatin demonstrated a more uniform and homogeneous crosslinking pattern, while sea bass gelatin developed a particulate and heterogeneous network. These differences are attributed to species-specific variations in amino acid composition and polypeptide chain organization, in agreement with previous reports indicating that gelatin properties are strongly dependent on fish species [12].

## 4. Conclusions

This study provides a comprehensive evaluation of transglutaminase (TG)-mediated crosslinking of gelatins derived from trout and sea bass skins, revealing significant effects on their structural, physicochemical, rheological, and functional properties. TG treatment notably enhanced gel strength, particularly in sea bass gelatin, supporting the formation of robust gel matrices with Bloom values up to 163 g. Rheological analyses confirmed improved viscoelasticity in a TG concentration-dependent manner, while functional properties such as emulsion activity, stability, and foaming capacity were modulated by TG, highlighting its role in regulating surface-active behavior. Spectroscopic analyses (UV–Vis and FTIR) indicated subtle TG-induced modifications in protein conformation, especially within Amide I and II regions, without disrupting the overall protein structure. Zeta potential measurements revealed significant changes in surface charge, reflecting enhanced colloidal stability, while thermal analyses demonstrated improved heat resistance, particularly in trout gelatins. Optical properties, including transparency and color, were also influenced by gelatin type and TG concentration, with trout gelatin exhibiting higher transparency and structural homogeneity, whereas sea bass gelatin formed a more stable and less variable network. Overall, these findings demonstrate that TG-modified fish gelatins possess tunable structural and functional characteristics, supporting their potential as versatile biopolymers for food, biomaterials, and packaging applications. Future research should address long-term stability and bioactive delivery capabilities to further expand their practical utility.

## Figures and Tables

**Table 1:** Physicochemical, functional and structural properties of trout, sea bass and TG-modified gelatins.

Samples	Transparency (%)	OBC (mL/g)	T_d_ (°C)
Trout gelatin	99.61 ± 0.13 ^bC^	16.06 ± 0.25 ^aA^	84.20 ± 0.14 ^aA^
T-TG0.01%	100.00 ^aA^	11.97 ± 0.41 ^bB^	77.12 ± 0.28 ^bB^
T-TG0.02%	100.00 ^aA^	11.00 ± 0.92 ^bBC^	120.95 ± 0.37 ^cC^
T-TG0.04%	100.00 ^Aa^	7.62 ± 0.17 ^cE^	114.40 ± 0.26 ^dD^
T-TG0.06%	99.31 ^cD^	10.61 ± 1.59 ^bcBCD^	72.24 ± 0.21 ^eE^
T-TG0.08%	100.00 ^aA^	10.99 ± 0.11 ^bBC^	127.70 ± 0.33 ^fF^
SB gelatin	99.54 ± 0 ^yC^	8.97 ± 0.32 ^xyCDE^	120.89 ± 0.45 ^xC^
SB-TG0.01%	99.77 ± 0 ^xB^	7.95 ± 0.33 ^xyDE^	131.23 ± 0.53 ^yG^
SB-TG0.02%	99.31 ± 0 ^zD^	6.8 ± 0.89 ^yE^	134.45 ± 0.20 ^zH^
SB-TG0.04%	99.31 ± 0 ^zD^	8.52 ± 0.06 ^xyCDE^	130.87 ± 0.15 ^tI^
SB-TG0.06%	99.31 ± 0 ^zD^	9.2 ± 0.89 ^xCDE^	133.27 ± 0.27 ^kK^
SB-TG0.08%	99.31 ± 0 ^zD^	7.07 ± 0.21 ^xyE^	111.51 ± 0.33 ^mM^

Data are expressed as mean ± standard deviation of triplicate determinations. Means with different lowercase letters in the same column indicate the statistical differences (*p* < 0.05) within the gelatins and TG-modified gelatins themselves, and different uppercase letters indicate statistical differences between all samples (*p* < 0.05). T: trout gelatin; SB: sea bass gelatin; TG: transglutaminase; OBC: oil-binding capacity; T_d_: denaturation temperature.

**Table 2:** Color properties of trout, sea bass and TG-modified gelatins.

Samples	L*	a*	b*	ΔE
Trout gelatin	100.58 ± 0.71 ^aA^	−3.29 ± 0.05 ^bE^	15.97 ± 0.27 ^bC^	-
T-TG0.01%	99.41 ± 0.70 ^abcABCD^	−3.02 ± 0.08 ^abCDE^	15.57 ± 0.32 ^bcC^	1.37 ± 1.36 ^aAB^
T-TG0.02%	100.61 ± 0.86 ^aA^	−2.82 ± 0.32 ^aABCD^	14.4 ± 1.05 ^cD^	1.65 ± 0.89 ^aAB^
T-TG0.04%	99.91 ± 0.35 ^abABC^	−3.1 ± 0.05 ^abDE^	18.15 ± 0.36 ^aAB^	2.30 ± 0.24 ^aAB^
T-TG0.06%	98.27 ± 0.10 ^cDE^	−2.91 ± 0.08 ^abBCD^	15.00 ± 0.26 ^bcCD^	1.41 ± 0.46 ^aAB^
T-TG0.08%	98.85 ± 0.26 ^bcBCDE^	−2.95 ± 0.09 ^abCD^	15.33 ± 0.33 ^bcCD^	2.23 ± 0.51 ^aAB^
SB gelatin	100.19 ± 0.41 ^xAB^	−2.53 ± 0.02 ^xA^	17.67 ± 0.13 ^zB^	-
SB-TG0.01%	97.97 ± 0.18 ^yDE^	−2.74 ± 0.02 ^ztABC^	18.48 ± 0.19 ^xyAB^	2.38 ± 0.54 ^xAB^
SB-TG0.02%	97.82 ± 0.21 ^yE^	−2.62 ± 0.02 ^yAB^	18.92 ± 0.16 ^xA^	2.69 ± 0.36 ^xA^
SB-TG0.04%	97.50 ± 0.17 ^yE^	−2.72 ± 0.01 ^czABC^	18.18 ± 0.11 ^yzAB^	2.74 ± 0.35 ^xA^
SB-TG0.06%	98.49 ± 0.59 ^yCDE^	−2.78 ± 0.02 ^tABC^	18.26 ± 0.26 ^yzAB^	0.60 ± 0.33 ^yB^
SB-TG0.08%	97.77 ± 0.70 ^yE^	−2.73 ± 0.03 ^ztABC^	18.28 ± 0.34 ^yAB^	0.80 ± 0.36 ^yB^

Data are expressed as mean ± standard deviation of triplicate determinations. Means with different lowercase letters in the same column indicate the statistical differences (*p* < 0.05) within the gelatin and TG-modified gelatins themselves, and different uppercase letters indicate statistical differences between all samples (*p* < 0.05). T: trout gelatin; SB: sea bass gelatin; TG: transglutaminase.

**Table 3:** Secondary structure percentage (%) analysis of trout, sea bass and TG-modified gelatins by analyzing the areas of 1600–1700 cm^−1^ in ATR-FTIR spectra.

Samples	β-Sheet	Random Coil	α-Helix	β-Turn
Trout gelatin	40.69 ± 0.07 ^abDE^	8.57 ± 0.57 ^aH^	10.06 ± 0.82 ^aC^	40.66 ± 0.52 ^aB^
T-TG0.01%	40.02 ± 0.92 ^bE^	12.97 ± 0.76 ^bB^	9.36 ± 0.89 ^bD^	37.63 ± 0.22 ^bE^
T-TG0.02%	37.23 ± 0.65 ^cF^	15.98 ± 0.61 ^cA^	12.00 ± 0.44 ^cB^	34.77 ± 0.68 ^cH^
T-TG0.04%	41.29 ± 0.87 ^aD^	8.66 ± 0.27 ^aH^	10.62 ± 0.86 ^aC^	39.40 ± 0.97 ^dC^
T-TG0.06%	40.03 ± 0.83 ^abE^	9.83 ± 0.35 ^dF^	9.25 ± 0.85 ^bD^	40.88 ± 0.38 ^aB^
T-TG0.08%	29.46 ± 0.43 ^dG^	11.91 ± 1.00 ^eCD^	12.15 ± 0.22 ^cB^	46.47 ± 0.53 ^eA^
SB gelatin	53.38 ± 0.80 ^xA^	12.13 ± 0.83 ^xC^	16.17 ± 0.74 ^xA^	18.29 ± 0.61 ^xJ^
SB-TG0.01%	43.06 ± 0.60 ^zC^	11.94 ± 0.97 ^xyCD^	9.09 ± 0.91 ^yD^	35.88 ± 0.50 ^yG^
SB-TG0.02%	40.83 ± 0.67 ^tDE^	11.73 ± 0.52 ^yD^	10.53 ± 0.61 ^zC^	36.89 ± 0.18 ^zF^
SB-TG0.04%	42.46 ± 0.31 ^kC^	10.46 ± 0.63 ^zE^	10.36 ± 0.9 ^zC^	36.70 ± 0.81 ^zF^
SB-TG0.06%	46.42 ± 0.65 ^yB^	9.17 ± 0.43 ^tG^	10.46 ± 0.58 ^zC^	33.93 ± 0.32 ^tI^
SB-TG0.08%	45.78 ± 0.63 ^yB^	8.58 ± 0.36 ^kH^	7.45 ± 0.60 ^tE^	38.18 ± 0.29 ^kD^

Data are expressed as mean ± standard deviation of triplicate determinations. Means with different lowercase letters in the same column indicate the statistical differences (*p* < 0.05) within the gelatin and TG-modified gelatins themselves, and different uppercase letters indicate statistical differences between all samples (*p* < 0.05). T: trout gelatin; SB: sea bass gelatin; TG: transglutaminase.

## Data Availability

The original contributions presented in this study are included in the article. Further inquiries can be directed to the corresponding author.

## Abbreviations

The following abbreviations are used in this manuscript:

T	Trout gelatin
SB	Sea bass gelatin
TG	Transglutaminase
DSC	Differential scanning calorimetry
OBC	Oil-binding capacity
EAI	Emulsion activity index
ESI	Emulsion stability index
PBS	Phosphate-buffered saline
SDS	Sodium dodecyl sulfate
SEM	Scanning electron microscopy
T_d_	Phase transition temperature
TUIK	Turkish Statistical Institute
FC	Foam Capacity
FS	Foam Stability
